# Assisted Dying, Pharmacological Lethality, and Suicide Risk Among Healthcare Workers: Ethical, Regulatory, and Mental Health Dimensions

**DOI:** 10.7759/cureus.103876

**Published:** 2026-02-18

**Authors:** Madhusudan P Singh, Pugazhenthan T

**Affiliations:** 1 Pharmacology and Therapeutics, Shri Shankaracharya Institute of Medical Sciences, Bhilai, IND; 2 Pharmacology and Therapeutics, All India Institute of Medical Sciences Raipur, Raipur, IND

**Keywords:** assisted dying, awareness, doctors psychological stress, healthcare workers, medical ethics, mental health issues, physician-assisted dying, professional burnout, psychological stress, suicide prevention

## Abstract

The intersection of assisted dying and suicides among healthcare workers presents a complex and multifaceted challenge, blending ethical, regulatory, and mental health considerations. This manuscript explores the bipolar association between these two phenomena, focusing on how healthcare professionals, particularly those with access to potent pharmacological agents, navigate the fine line between assisted dying in clinical settings and the alarming trend of suicides within the medical community. The ethical dilemmas posed by medical assistance in dying (MAiD) protocols are examined, including the responsibilities of healthcare providers, the delicate balance of patient autonomy versus societal norms, and the safeguarding of vulnerable populations. In parallel, the manuscript delves into the concerning rise of suicides among healthcare workers, often exacerbated by workplace stress, mental health challenges, and easy access to lethal medications. Recent post-pandemic data highlight moral injury, workforce attrition, and expanding MAiD eligibility criteria as emerging stressors that may further compound suicide risk among healthcare professionals. A detailed analysis of pharmacological agents commonly used in both MAiD and suicides highlights the regulatory measures needed to prevent misuse. Case studies illustrate the overlap between assisted dying and healthcare worker suicides, offering insights into the psychological, emotional, and systemic pressures faced by medical professionals. The manuscript calls for stronger mental health support systems within the healthcare community and advocates for regulatory reforms to ensure that assisted dying protocols are not exploited. Ultimately, it emphasizes the need for a nuanced approach to both assisted dying and healthcare worker suicide prevention, promoting ethical frameworks that respect patient rights while protecting the mental well-being of those who provide care.

## Introduction and background

Assisted dying, also referred to as medical assistance in dying (MAiD), denotes legally regulated medical practices in which clinicians prescribe or administer medications to intentionally hasten death at a patient’s request, most commonly in the context of terminal or refractory illness [1]. As an increasing number of jurisdictions move toward legalization, assisted dying has gained global relevance; however, its intersection with the mental health of healthcare workers directly involved in assisted dying and end-of-life care, who may have authorized access to highly lethal medications, remains insufficiently examined.

Although assisted dying practices are designed to alleviate patient suffering, they also place involved healthcare professionals in sustained proximity to death, complex ethical decision-making, and repeated handling of life-ending pharmacological agents. Healthcare professionals are known to experience a consistently higher risk of suicide than the general population, a vulnerability associated with occupational stress, moral injury, and access to lethal means [2]. This convergence, where the same pharmacological agents are used both to facilitate patient death and, in some cases, self-harm by clinicians, raises important questions regarding occupational vulnerability, access-to-means, and the mental well-being of healthcare workers engaged in end-of-life care.

Healthcare workers are routinely exposed to death, suffering, and ethically complex decision-making [3]. Clinicians directly involved in end-of-life care and assisted dying practices, where legally permitted, operate in settings characterized by heightened ethical responsibility, emotional labor, and professional exposure to death [4]. Although existing regulations are designed to ensure that assisted dying remains a tightly controlled, patient-centered process, the cumulative psychological burden borne by healthcare professionals, who already experience disproportionately higher suicide rates than the general population, often receives inadequate attention. At present, empirical data quantifying suicide rates specifically among clinicians involved in assisted dying remain limited. Consequently, this review does not seek to establish prevalence estimates, but rather to examine plausible occupational vulnerabilities and ethical concerns based on available literature.

Exposure to ethically complex and emotionally demanding clinical scenarios has been associated with moral distress and psychological strain among clinicians, which may contribute to mental health vulnerability in certain occupational contexts, and is a well-recognized and potentially modifiable risk factor for suicide [5]. Pharmacological agents commonly used in assisted dying, including barbiturates, opioids, and sedative-hypnotics, are also frequently implicated in suicides among healthcare workers [6]. Repeated professional familiarity with these agents, alongside moral distress and emotional exhaustion, may inadvertently lower psychological barriers to self-harm, blurring the distinction between therapeutic, palliative, and lethal use. This convergence underscores the urgent need for targeted safeguards for clinicians working in environments where life-ending drugs are readily accessible.

This manuscript examines the intersection between assisted dying and suicide risk among healthcare workers, situating this relationship within ethical, legal, pharmacological, and mental health frameworks. Rather than addressing assisted dying or healthcare worker suicide as isolated phenomena, the review focuses on their potential overlap as an under-recognized occupational and ethical concern.

The scope of this review is intentionally restricted to clinicians who are directly involved in assisted dying and closely related end-of-life care, where such practices are legally permitted. These clinicians operate at the convergence of repeated exposure to death, responsibility for ethically complex end-of-life decisions, and professional access to highly lethal medications. Each of these factors has been independently associated with psychological strain and suicide vulnerability among healthcare professionals, although empirical data specifically quantifying suicide risk among clinicians involved in assisted dying remain limited.

Within this context, the manuscript explores the pharmacological overlap between medications used in assisted dying, such as sedatives, opioids, and barbiturates, and those implicated in suicides among healthcare workers. Attention is given to occupational access to these agents as a potential risk modifier, without implying causality, and acknowledging the current gaps in direct epidemiological evidence.

The review further examines the mental health challenges encountered by clinicians engaged in assisted dying and end-of-life care, including moral distress, emotional burden, and ethical tension, situating these experiences within existing regulatory and professional frameworks. Participation in assisted dying is recognized as voluntary in most jurisdictions, and the manuscript does not assume coercion or universal distress among participating clinicians.

Finally, this review discusses preventive strategies relevant to this specific professional subgroup, emphasizing controlled medication access, institutional safeguards, voluntary participation policies, and targeted mental health support systems. By foregrounding clinician well-being within assisted dying frameworks, the manuscript advocates for ethical and regulatory approaches that safeguard healthcare workers while preserving patient autonomy.

## Review

Literature identification approach

This manuscript was conceived as a narrative, concept-driven review examining the interface between assisted dying and suicide risk among healthcare workers. Relevant literature was identified through targeted searches of PubMed, Scopus, and Embase using combinations of keywords related to assisted dying, medical assistance in dying, healthcare worker suicide, moral injury, pharmacological protocols, and ethical and regulatory frameworks. Priority was given to peer-reviewed articles, policy documents, and authoritative reports published between 2018 and 2024 that directly informed the ethical, clinical, pharmacological, and governance-related themes of the review. As this work is intended as a narrative, concept-driven review rather than a systematic or scoping review, formal procedures such as exhaustive search strategies, predefined inclusion and exclusion criteria, and risk-of-bias assessments were not applied. The emphasis was on thematic synthesis and interdisciplinary interpretation to explore ethical, pharmacological, and occupational dimensions of assisted dying relevant to healthcare worker vulnerability.

Global legal and policy perspectives on assisted dying

The United Nations Commission on Narcotic Drugs (CND) convened its second meeting in December 2023 to review the implementation of international drug control treaties, with particular attention to evolving challenges in the regulation and scheduling of controlled substances [7]. Discussions highlighted the growing complexity faced by the Commission, the World Health Organization (WHO), and the International Narcotics Control Board (INCB) in balancing public health priorities with effective control mechanisms, especially for substances with legitimate medical applications.

At the global level, drug control policies are increasingly shaped by the need to establish regulatory frameworks that both ensure access to essential medicines and prevent diversion or misuse. These frameworks involve the scheduling of controlled substances, oversight of their manufacture and distribution, and regulation of their clinical use for legitimate medical and scientific purposes [8,9]. In the context of assisted dying and palliative care, this balance is particularly delicate, as medications with high lethality are simultaneously indispensable for end-of-life care and vulnerable to misuse when safeguards are inadequate.

Contemporary international discourse has shifted away from purely enforcement-driven approaches toward integrated, public health-oriented models of drug governance. Alongside regulatory oversight, countries are encouraged to adopt demand-reduction strategies that include evidence-based prevention programs, accessible treatment and rehabilitation services, and social reintegration initiatives [9]. Importantly, recent policy emphasis has underscored the ethical obligation to ensure the availability of controlled substances for pain relief and end-of-life care, while implementing proportionate safeguards to prevent diversion, occupational misuse, and self-harm among healthcare professionals with authorized access to these agents (Table 1).

**Table 1:** Impact of Assisted Dying Legalization on Healthcare Systems Note: These impacts vary across different healthcare systems and cultural contexts. Source: [10-13]

Aspect	Observed Changes	Challenges	Opportunities
Palliative care	Generally improved	Resource allocation	Enhanced end-of-life care options
Healthcare costs	Mixed findings	Potential pressure for cost-saving	Reduced futile treatments
Doctor-patient relationship	Increased end-of-life discussions	Potential distrust	Improved communication
Medical education	Curriculum changes	Ethical dilemmas	Comprehensive end-of-life training
Healthcare worker stress	Increased in some studies	Moral distress	Professional development in ethics
Public health	Increased awareness of end-of-life issues	Stigma and misinformation	Improved advanced care planning

Global legislative landscape and perspectives

While early debates on assisted dying largely centered on Western jurisdictions, an increasing number of regions worldwide are now engaging with its legal, ethical, and cultural implications. These developments reflect not only evolving societal attitudes toward autonomy and end-of-life care but also growing recognition of the safeguards required to protect vulnerable patients and healthcare professionals involved in assisted dying practices.

European Perspective

Countries such as the Netherlands and Belgium have legalized assisted dying since the early 2000s and are often cited as models for regulated implementation [14]. These jurisdictions have established structured oversight mechanisms emphasizing voluntariness, proportionality, and the integration of palliative care [15]. In the Netherlands, euthanasia accounts for approximately 4.5% of all deaths, predominantly among elderly patients and those with terminal illnesses [16]. Ongoing monitoring has highlighted both procedural robustness and emerging ethical challenges related to case volume, clinician burden, and societal normalization.

Belgium permits euthanasia for psychiatric conditions under stringent eligibility criteria, a policy that continues to provoke ethical debate regarding decisional capacity, chronic suffering, and the adequacy of safeguards for vulnerable individuals [17]. Recent scholarly and regulatory discussions increasingly emphasize the psychological and moral implications for clinicians tasked with evaluating and performing such requests.

Switzerland presents a distinct legal model in which assisted suicide is permitted provided there is no selfish motive, with organizations such as Dignitas and Exit playing a prominent role [18]. This approach has influenced broader European discourse and prompted renewed examination of assisted dying legislation in neighboring countries, including Germany, where recent debates reflect shifting societal attitudes alongside continued judicial and ethical caution [19].

North America

In the United States, several states, including Oregon, Washington, and California, have enacted Death with Dignity statutes allowing terminally ill patients to self-administer prescribed life-ending medications [20]. Utilization has steadily increased; in California alone, more than 3,000 individuals accessed this option by 2023, indicating growing societal acceptance and institutional normalization of assisted dying [21].

Canada has implemented one of the most expansive MAiD frameworks since its introduction in 2016 [22]. Although eligibility was broadened in 2021 to include individuals whose sole underlying condition is mental illness, implementation of this provision has been repeatedly deferred amid concerns regarding safeguards, clinician preparedness, and the potential impact on vulnerable populations [22]. These delays underscore persistent ethical uncertainty and highlight the importance of balancing access with professional, institutional, and societal readiness.

Asia’s Emerging Discussion

Across Asia, assisted dying remains legally prohibited in most jurisdictions, yet public and professional discourse is increasing, often against a backdrop of rising suicide rates and demographic ageing. In Japan, sustained concern over suicide prevalence has stimulated debate regarding end-of-life autonomy, although strong cultural norms emphasizing family involvement and collective decision-making continue to limit legislative momentum [23].

In South Korea, parliamentary discussions have considered euthanasia-related legislation, particularly in response to an ageing population and increasing recognition of mental health needs [24]. Despite advances in palliative care and psychosocial services, euthanasia remains contentious, reflecting broader societal ambivalence toward intentional life-ending interventions.

Latin America and Africa

Colombia remains the most prominent example in Latin America, having legalized euthanasia in 1997 and subsequently expanding access under defined regulatory frameworks [25]. Its experience illustrates how assisted dying can be integrated within a predominantly religious society through judicial oversight and procedural safeguards. Elsewhere in the region, countries such as Argentina and Brazil continue to prohibit assisted dying, although advocacy efforts and ethical debate are gaining visibility [26].

In Africa, discussions surrounding assisted dying are shaped by cultural values, resource limitations, and competing public health priorities. Many countries prioritize expanding access to basic healthcare and palliative services over legislative consideration of euthanasia. South Africa has initiated public and legal debates on assisted dying, yet progress remains constrained by ethical pluralism and systemic healthcare challenges. In nations such as Zimbabwe and Kenya, euthanasia remains taboo, further complicating discourse on end-of-life autonomy and clinician responsibilities (Table 2) [26,27].

**Table 2:** Comparative Analysis of Assisted Dying Safeguards Across Jurisdictions *With parental consent until 16, then independent decision from 16-18. Source: [28,29]

Safeguard Measure	Netherlands	Belgium	Oregon (USA)	Canada	Switzerland
Minimum age	12*	No minimum	18	18	18
Waiting period	1-14 days	1 month	15 days	10 days	None specified
Second opinion	Required	Required	Required	Required	Not always required
Psychiatric evaluation	If capacity in doubt	Required for non-terminal cases	If capacity in doubt	If capacity in doubt	If capacity in doubt
Reporting requirement	Mandatory	Mandatory	Mandatory	Mandatory	Varies by organization
Non-residents eligible	No	Yes	No	No	Yes

Indian Perspective

In India, the debate surrounding assisted dying and the decriminalization of suicide has intensified in recent years, reflecting a broader re-examination of end-of-life rights, mental health legislation, and individual autonomy [30]. This discourse is shaped by recurring judicial engagement, particularly by the Supreme Court, on the constitutional right to die with dignity, and is deeply influenced by cultural values, ethical considerations, and societal expectations. Together, these factors underscore the complexity of formulating coherent end-of-life policies within the Indian legal framework.

A major legislative development is reflected in the proposed repeal of Section 309 of the Indian Penal Code (IPC), effectively decriminalizing attempted suicide and signaling a shift toward a more compassionate, health-centered approach to mental illness [31,32]. This reform acknowledges suicide attempts as manifestations of psychological distress rather than criminal intent. However, the introduction of Section 224 preserves criminal liability in cases where suicide attempts are undertaken to obstruct public servants in the discharge of their official duties, reflecting the state’s effort to deter extreme forms of protest [31,32].

The enactment of Section 115 of the Mental Healthcare Act (MHCA), 2017, marked a further milestone by presuming severe stress in individuals who attempt suicide and mandating care rather than punishment [33]. Nevertheless, the simultaneous existence of Section 309 in the IPC until recent legislative reforms created legal ambiguity, which in practice exposed vulnerable individuals to inconsistent enforcement and occasional harassment [33]. While procedural protocols were subsequently established for the medical and psychiatric management of suicide attempt survivors, the implementation of these safeguards has remained uneven across healthcare settings, highlighting systemic gaps in mental health infrastructure and follow-up care.

Parallel to suicide decriminalization, India’s legal position on euthanasia has evolved through judicial intervention rather than comprehensive legislation. In its landmark 2018 judgment, the Supreme Court recognized the right to die with dignity and permitted passive euthanasia under specified conditions, introducing guidelines for advance directives and living wills [34]. Subsequent clarifications have sought to simplify these procedures; however, practical challenges persist, particularly due to limited clinician training, inconsistent institutional ethics committees, and low public awareness of palliative and end-of-life care options.

From a clinical and ethical standpoint, there is an urgent need to strengthen training in palliative care, end-of-life ethics, and shared decision-making for healthcare professionals. Expanding access to palliative care services and establishing dedicated units within hospitals can improve symptom control, psychosocial support, and informed end-of-life planning, thereby reducing reliance on legally and ethically complex interventions.

India’s narcotics regulatory framework, governed by the Narcotic Drugs and Psychotropic Substances (NDPS) Act, 1985, plays a critical role in regulating medications used in palliative care and potential assisted dying contexts [35]. Recent amendments have aimed to improve access to essential controlled medicines for patients with terminal illnesses while maintaining safeguards against diversion and misuse. However, these reforms also place significant responsibility on healthcare professionals, underscoring the importance of structured training in the legal, ethical, and practical aspects of handling controlled substances.

Strengthening institutional oversight, improving clinician education, and integrating mental health support for healthcare workers involved in end-of-life care are essential to ensure that evolving legal reforms protect both patient dignity and the psychological well-being of healthcare professionals entrusted with administering life-ending or life-limiting therapies.

Mental health vulnerability and suicide risk in healthcare workers

The persistently elevated rates of suicide among healthcare professionals underscore an urgent need for targeted and proactive mental health interventions within this workforce. Healthcare workers face a unique constellation of occupational stressors, including repeated exposure to death, high-stakes decision-making, moral and ethical conflict, and, in some cases, direct participation in end-of-life care. For clinicians involved in assisted dying or palliative sedation, the psychological burden may be particularly pronounced, increasing vulnerability to emotional exhaustion, moral injury, and suicidal ideation.

Effective suicide prevention in healthcare settings requires approaches that move beyond generic wellness initiatives toward strategies tailored to occupational risk. Institutions must prioritize the availability of confidential, easily accessible mental health services specifically designed for healthcare professionals, including counselling, psychological first aid, and structured support following exposure to distressing clinical events. Creating psychologically safe workplaces, where discussions of distress are normalized and help-seeking is not perceived as professional weakness, is central to reducing stigma and encouraging early intervention.

Education and training play a critical role in prevention. Regular, structured training programs that enable healthcare workers and supervisors to recognize early signs of psychological distress, moral injury, and burnout can facilitate timely support. Peer support models, particularly those integrated into clinical teams, have shown promise in fostering resilience, reducing isolation, and providing informal pathways to care. Such interventions not only protect the mental well-being of healthcare professionals but also contribute to improved patient care, especially in emotionally complex end-of-life contexts.

At the global level, suicide prevention among healthcare workers has gained increasing recognition as a public health priority. The World Health Organization (WHO) has advocated comprehensive, multi-level strategies to reduce suicide risk among vulnerable populations, emphasizing mental health promotion, early identification of risk, and improved access to care [36]. International experience demonstrates the effectiveness of coordinated prevention frameworks: countries such as Finland and Australia have implemented national programs emphasizing community engagement, crisis intervention, and continuity of care following suicide attempts [37]. Similarly, initiatives such as the WHO’s LIVE LIFEcampaign and the Thrive program in the United Kingdom illustrate how suicide prevention can be integrated into broader public health and workplace mental health strategies, offering valuable models for adaptation within healthcare systems [38-40]. Having outlined the global and legal landscape, the following section examines the pharmacological agents central to assisted dying and their relevance to healthcare worker suicide risk.

Pharmacological agents used in assisted dying and their dual-risk implications

The pharmacological approach to assisted dying involves the deliberate use of medication regimens designed to produce rapid unconsciousness followed by death, with the explicit aim of minimizing distress and suffering at the end of life [41]. These protocols typically employ combinations of sedative-hypnotics, opioids, and neuromuscular or cardiopulmonary depressant agents, selected for their predictable pharmacokinetics, reliability, and capacity to induce a peaceful death. A detailed overview of the commonly used agents, their mechanisms of action, dosing considerations, and roles within assisted dying protocols is summarized in Table 3 [41,42].

**Table 3:** Pharmacological Agents Utilized in Assisted Dying Procedures: Roles, Mechanisms, and Utilization in Medical Assistance in Dying (MAiD) MAiD, medical assistance in dying; CNS, central nervous system; GABA-A, gamma-aminobutyric acid type A receptor; NMBA, neuromuscular blocking agent; NMDA, N-methyl-D-aspartate receptor; μg, microgram; mg, milligram; g, gram; mEq, milliequivalent. *Onset of action refers to the approximate time to clinically significant sedation, unconsciousness, or pharmacodynamic effect following administration. Source: [41-43] *Utilization frequency reflects reported proportions of drug use within MAiD protocols where data are available.

Drug Class	Examples	Role in Assisted Dying	Mechanism of Action	Dosage/Utilization	Onset of action	Utilization Frequency in MAiD
Barbiturates	Pentobarbital, secobarbital, phenobarbital	Induces unconsciousness, respiratory depression, and cardiovascular collapse, leading to death.	Enhances GABA-A receptor activity, increasing chloride influx and causing CNS depression.	Pentobarbital: 1-15 g Secobarbital: 9 g Phenobarbital: 3000 mg	30-60 minutes	Phenobarbital: 0.1%
Benzodiazepines	Midazolam, diazepam, lorazepam	Sedation and anxiety reduction pre-procedure.	Enhances GABA-A receptor activity, promoting CNS depression and sedation.	Midazolam: 2-120 mg (median 10 mg) Diazepam: 10-120 mg Lorazepam: 2.5-5 mg	5-15 minutes	Midazolam: 91.4%
Neuromuscular blocking agents (NMBAs)	Rocuronium, cisatracurium, atracurium, pancuronium, vecuronium	Induces paralysis, ensuring no involuntary movements during the procedure.	Blocks acetylcholine at the neuromuscular junction, preventing muscle contraction.	Rocuronium: 50-300 mg (median 200 mg) Cisatracurium: 30-40 mg (median 40 mg) Atracurium: 50-100 mg Pancuronium: 18-20 mg Vecuronium: 10-60 mg	1-5 minutes	Rocuronium: 92.6% Cisatracurium: 9.1%
Opioids	Morphine, hydromorphone, fentanyl	Provides analgesia and sedation, while facilitating respiratory depression.	Binds to mu-opioid receptors, reducing pain perception and consciousness.	Morphine: 16-480 mg Fentanyl: 25-1500 μg	15-30 minutes	0.6% (specified opioids)
Anesthetics	Propofol, thiopental	Primary anesthetic inducing rapid and reversible unconsciousness, facilitating a controlled process.	Increases GABA-A receptor activity, leading to CNS depression and rapid unconsciousness.	Propofol: 1000-2000 mg (median 1000 mg) Thiopental: 1-2 g	3-5 minutes	Propofol: 99.0%
Analgesics	Lidocaine	Provides localized pain relief during the procedure.	Blocks sodium channels, inhibiting nerve signal transmission.	40-120 mg (median 40 mg)	45–90 seconds	81.7%
Cardiotoxic agents	Potassium chloride, bupivacaine	Induces cardiac arrest.	Disrupts normal heart rhythm and function.	Potassium chloride: median 80 mEq Bupivacaine: 400 mg	Varies	Potassium chloride: 4.0% Bupivacaine: 20.3%
Inhalational anesthetics	Nitrous oxide	Provides sedation and anesthesia, inducing unconsciousness	NMDA receptor antagonist; reduces pain perception.	20%-70%	2-5 minutes	-
Inhalational anesthetics	Ketamine	May provide pain relief and sedation, but poses risks of dissociative and hallucinogenic experiences.	NMDA receptor antagonist; disrupts normal pain perception.	For anaesthesia: 2mg/kg body weight	15-30 seconds	-
Others	Magnesium sulfate	Potentiates the effects of other drugs and may contribute to cardiorespiratory depression.	Multiple mechanisms including NMDA receptor antagonism and calcium channel blockade.	1000 mg	Varies	-

While pharmacological protocols for assisted dying are subject to regulatory oversight and clinical guidelines, recent trends toward standardization, particularly in jurisdictions with established MAiD frameworks, have increased clinician familiarity with these drug combinations. Although such standardization enhances procedural safety and consistency, repeated professional exposure to highly lethal medications may also carry unintended psychological consequences. Familiarity with the mechanisms, dosing, and lethality of these agents may lower cognitive and emotional barriers to self-harm among vulnerable healthcare workers, underscoring the importance of contextualizing pharmacological practices within broader occupational mental health safeguards.

Mental health dimensions and suicide prevention among healthcare workers

One of the most significant and under-recognized consequences of assisted dying practices is the psychological impact on healthcare professionals directly involved in end-of-life care. Clinicians participating in assisted dying frequently operate at the ethical and emotional boundary between palliation and the intentional hastening of death, often administering the same pharmacological agents used in both palliative sedation and assisted dying [44]. Continuous exposure to death, coupled with routine handling of potent life-ending medications, places these individuals at heightened risk for emotional exhaustion, moral injury, and psychological distress.

Healthcare professionals already experience higher rates of depression, burnout, and suicide compared with the general population [44]. Engagement in assisted dying procedures may intensify these vulnerabilities by adding layers of ethical tension, emotional responsibility, and potential internal conflict, even when actions align with legal and professional standards. Crucially, occupational access to highly lethal drugs, combined with repeated familiarity and desensitization, has been identified as a significant risk factor for suicide among healthcare workers [45]. This convergence of psychological strain and access to means highlights a critical gap in existing assisted dying frameworks, which often prioritize patient safeguards while insufficiently addressing clinician well-being.

Addressing these risks requires that mental health protections for healthcare workers be recognized as an integral component of assisted dying programs. Institutional responses must extend beyond individual resilience toward systemic preventive measures, including structured psychological support, monitoring of occupational stress, and safeguards around access to lethal medications [44,45]. While indicators of mental health vulnerability may allow early identification of clinicians at risk (Table 4), prevention requires system-level safeguards tailored to the unique ethical, pharmacological, and occupational features of assisted dying practice. Table 5 summarizes assisted dying-specific strategies aimed at mitigating suicide risk among clinicians, emphasizing institutional responsibility alongside individual support.

**Table 4:** Potential Indicators of Mental Health Risk in Healthcare Workers Involved in Assisted Dying Source: [44,45]

Indicator	Description	Recommended Action
Changes in behavior	Sudden mood changes, emotional blunting, or withdrawal following involvement in assisted dying or end-of-life decision-making	Confidential peer support, targeted psychological assessment following assisted dying involvement
Increased absenteeism	Frequent or unexplained absences after participation in assisted dying procedures or related ethical deliberations	Supportive, non-punitive inquiry; review of role burden and temporary modification of assisted dying duties
Decline in job performance	Increased errors, reduced concentration, or difficulty with complex decisions in clinical roles involving assisted dying	Occupational health review, mentoring, and workload adjustment within assisted dying responsibilities
Expressed feelings of hopelessness	Verbalized emotional distress, moral conflict, or existential pessimism linked to repeated exposure to death or ethically challenging cases	Immediate mental health referral and ethics-informed counselling
Substance misuse indicators	Increased reliance on alcohol or medications as coping mechanisms following emotionally distressing assisted dying cases	Early referral to employee assistance or addiction services, with attention to medication access
Increased interpersonal conflict	Heightened irritability or conflict with colleagues, patients, or families during or after assisted dying-related work	Facilitated team debriefing, conflict resolution support, and reflective practice sessions
Physical symptoms	Fatigue, sleep disturbance, or somatic complaints associated with anticipatory stress or post-procedure rumination	Health evaluation, stress-management interventions, and scheduled recovery time following assisted dying involvement

**Table 5:** Assisted dying–specific strategies to mitigate suicide risk among clinicians Source: [46-57]

Domain	Assisted Dying–Specific Risk Context	Targeted Preventive Strategy
Medication access and handling	Clinicians involved in assisted dying have professional access to highly lethal medications (e.g., barbiturates, sedatives, opioids) used for both patient death and self-harm	Restricted access protocols for assisted dying drugs, dual authorization systems, controlled storage, audit trails, and separation of prescribing and dispensing responsibilities
Ethical and moral burden	Participation in assisted dying may involve moral distress, ethical uncertainty, or conflict with personal values	Structured ethics consultation services, regular ethics debriefings, and access to moral injury–informed counselling for clinicians involved in assisted dying
Psychological exposure to death	Repeated involvement in end-of-life decisions and witnessing death may contribute to emotional exhaustion	Mandatory post-procedure psychological debriefing, reflective practice sessions, and peer-led discussion forums specific to assisted dying cases
Voluntariness of participation	Clinicians may feel implicit professional pressure despite legal voluntariness	Explicit institutional policies reaffirming voluntary participation, clear opt-out mechanisms, and protection from professional disadvantage for non-participation
Occupational isolation	Assisted dying roles may be undertaken by a limited group of clinicians, increasing professional and emotional isolation	Designated assisted dying teams with rotating responsibilities, peer support networks, and mentorship structures
Training and preparedness	Inadequate preparation may amplify distress during assisted dying procedures	Specialized training covering ethical decision-making, communication around assisted dying, emotional resilience, and self-monitoring for distress
Early identification of distress	Clinicians involved in assisted dying may normalize distress or hesitate to seek help	Periodic confidential mental health screening for clinicians involved in assisted dying, with clear referral pathways
Post-event support	Psychological impact may emerge after assisted dying cases rather than immediately	Structured follow-up support at predefined intervals following assisted dying involvement
Institutional culture	Stigma around clinician vulnerability may be heightened in ethically sensitive practices	Leadership-driven normalization of help-seeking, explicit acknowledgment of emotional impact of assisted dying work
Policy and regulation	Assisted dying frameworks often prioritize patient safeguards over clinician well-being	Integration of clinician mental health safeguards into assisted dying legislation, institutional protocols, and regulatory oversight

Preventive strategies to reduce suicide risk among healthcare workers

Effective prevention of suicide among healthcare workers involved in assisted dying requires a multi-layered approach that integrates pharmacological safeguards, institutional responsibility, and proactive mental health support [30,46-48]. Given the dual realities of occupational stress and access to highly lethal medications, preventive strategies must extend beyond individual-level interventions to encompass structural and governance-based protections.

Robust controls over access to medications used in assisted dying are essential to minimize the risk of diversion and self-harm. Restricting access to authorized personnel directly involved in assisted dying procedures is a foundational measure. Such restrictions should be reinforced through dual authorization protocols, ensuring that dispensing and administration of lethal medications require independent verification by more than one qualified professional [49,50].

Secure, designated storage facilities with restricted entry are critical for limiting unauthorized access. Incorporation of biometric access systems, such as fingerprint or retina-based authentication, provides an additional layer of protection by ensuring that only credentialed individuals can access controlled substances. Complementing these measures, electronic logging systems should be employed to document all instances of drug access and handling, enabling real-time tracking and facilitating regular audits to identify discrepancies or misuse.

Structural safeguards alone are insufficient without parallel investment in healthcare worker mental well-being. Institutions must establish confidential, non-punitive mental health services tailored specifically for healthcare professionals involved in end-of-life care. These services should include psychological counselling, stress management programs, and targeted interventions following participation in assisted dying procedures [51-54].

Periodic mental health assessments for healthcare workers with access to lethal medications can help identify early signs of distress, burnout, or moral injury. Such assessments should be framed as supportive rather than evaluative to avoid stigmatization and ensure engagement. Peer support programs, particularly those embedded within clinical teams, offer an accessible and trusted avenue for emotional support and early help-seeking.

Training and certification programs are also vital. Mandatory education on the ethical, legal, and psychological dimensions of assisted dying, including awareness of suicide risk factors and self-care strategies, can empower healthcare professionals to recognize vulnerability in themselves and colleagues. Establishing protected spaces for ethical reflection and debriefing following assisted dying cases may further mitigate cumulative emotional burden.

Sustainable prevention requires strong institutional governance and external oversight. Regular audits and reviews of medication inventories, access logs, and assisted dying cases are essential to ensure compliance with established protocols. Discrepancies must trigger timely investigation and corrective action.

Independent oversight bodies should be tasked with monitoring adherence to assisted dying regulations, evaluating safeguards for healthcare worker well-being, and reviewing adverse events, including suspected misuse of medications. Collaboration with regulatory authorities enhances accountability and ensures alignment with national legal frameworks governing controlled substances [55-57].

The establishment of whistle-blower protection mechanisms is equally important. Healthcare workers must be able to report concerns related to medication handling, unsafe practices, or colleague distress without fear of retaliation. Transparent reporting pathways foster a culture of safety, ethical practice, and shared responsibility [49-57].

## Conclusions

As assisted dying becomes an established component of end-of-life care in many jurisdictions, ethical and regulatory frameworks must evolve to address not only patient protections but also the well-being of clinicians who participate in these practices. End-of-life decision-making places unique emotional, ethical, and professional demands on healthcare workers, particularly in settings involving routine access to highly lethal medications.

Sustainable assisted dying systems require safeguards that extend beyond procedural compliance. Policies should ensure voluntary participation, controlled medication access, confidential mental health support, and institutional cultures that acknowledge the psychological impact of end-of-life care. Embedding clinician well-being into assisted dying governance strengthens ethical integrity, supports workforce sustainability, and reduces preventable harm. Balancing patient autonomy with the mental health and safety of healthcare professionals is essential for the long-term legitimacy and humane implementation of assisted dying practices. Proactive, system-level approaches that integrate clinician protection alongside patient safeguards will be critical as assisted dying frameworks continue to expand globally.
